# Nanobiosensors for the Detection of Novel Coronavirus 2019-nCoV and Other Pandemic/Epidemic Respiratory Viruses: A Review

**DOI:** 10.3390/s20226591

**Published:** 2020-11-18

**Authors:** Badriyah Alhalaili, Ileana Nicoleta Popescu, Olfa Kamoun, Feras Alzubi, Sami Alawadhia, Ruxandra Vidu

**Affiliations:** 1 Nanotechnology and Advanced Materials Program, Kuwait Institute for Scientific Research, P.O. Box 24885, Safat 13109, Kuwait; bhalaili@kisr.edu.kw (B.A.); fzubi@kisr.edu.kw (F.A.); sawadhi@kisr.edu.kw (S.A.); 2 Faculty of Materials Engineering and Mechanics, Valahia University of Targoviste, 13 Aleea Sinaia Street, 130004 Targoviste, Romania; 3 Physics of Semiconductor Devices Unit, Faculty of Sciences of Tunis, Tunis El Manar University, Tunis 1068, Tunisia; o.kamoun@yahoo.fr; 4 Faculty of Materials Science and Engineering, University Politehnica of Bucharest, 060042 Bucharest, Romania; 5 Department of Electrical and Computer Engineering, University of California Davis, Davis, CA 95616, USA

**Keywords:** nanobiosensors, COVID-19 pandemic, coronavirus 2019-nCoV, SARS/MERS-CoV, influenzas, respiratory virus, virus detection, nanomaterials

## Abstract

The coronavirus disease 2019 (COVID-19) pandemic is considered a public health emergency of international concern. The 2019 novel coronavirus (2019-nCoV) or severe acute respiratory syndrome coronavirus 2 (SARS-CoV-2) that caused this pandemic has spread rapidly to over 200 countries, and has drastically affected public health and the economies of states at unprecedented levels. In this context, efforts around the world are focusing on solving this problem in several directions of research, by: (i) exploring the origin and evolution of the phylogeny of the SARS-CoV-2 viral genome; (ii) developing nanobiosensors that could be highly effective in detecting the new coronavirus; (iii) finding effective treatments for COVID-19; and (iv) working on vaccine development. In this paper, an overview of the progress made in the development of nanobiosensors for the detection of human coronaviruses (SARS-CoV, SARS-CoV-2, and Middle East respiratory syndrome coronavirus (MERS-CoV) is presented, along with specific techniques for modifying the surface of nanobiosensors. The newest detection methods of the influenza virus responsible for acute respiratory syndrome were compared with conventional methods, highlighting the newest trends in diagnostics, applications, and challenges of SARS-CoV-2 (COVID-19 causative virus) nanobiosensors.

## 1. Introduction

As a result of the coronavirus disease 2019 (COVID-19) pandemic, most research efforts around the world are focusing on solving this pressing problem, firstly, by developing ultrafast detection of the virus and isolating infected people, and secondly, finding and developing effective treatments, including vaccines specific to this new disease.

Consequently, the world is facing a new challenge: to develop ultra-rapid, ultra-sensitive devices, and nanoscale analytical tools, or sensing systems (e.g., nanobiosensors) that are highly effective at detecting the 2019 novel coronavirus (2019-nCoV) or severe acute respiratory syndrome (SARS) coronavirus-2 (SARS-CoV-2) [1,2,3,4,5,6,7] associated with the new disease, broadly referred to as COVID-19 [8], CO for corona (Latin: corona = crown), VI for virus, D for disease, and 2019 for the year this novel disease appeared. This is the third major epidemic based on severe acute respiratory syndrome in the last 20 years.

The previous coronavirus epidemics, severe acute respiratory syndrome (SARS) and Middle East respiratory syndrome (MERS), are closely related to the 2002 SARS virus (SARS-CoV), with symptoms similar to pneumonia or the flu (influenza infections) and Middle East respiratory syndrome coronavirus (MERS-CoV).

The infection with MERS-CoV, SARS Coronavirus-2, or SARS-CoV, causes severe and often lethal lung failure [9], the differences consisting mainly in transmission rate, incubation period, and case fatality rate [8]. Since March 2020, more than 80 countries have released new safety regulations, such as closing the borders to highly-infected countries, closing businesses, requiring self-quarantine, and closing schools, shopping centers, and governmental institutes. The disease is destructively changing global economic growth. This global health and economic crisis has affected the global economy by over $90 trillion [10], which has not happened in almost a century. The new virus can target millions of people, especially those who suffer from numerous medical problems [3,5,11,12]. Unfortunately, the cause of novel coronavirus 2019 (SARS-CoV 2 or 2019-nCoV) is unknown. We are aware of its transmission to others by direct contact via respiratory droplets of an infected person (generated through coughing and sneezing) or by touching surfaces contaminated with the virus, and then touching their faces (e.g., eyes, nose, mouth). Consequently, it is extremely important to avoid virus transmission [13] and learn how to reduce its impact on people all over the world.

Precautions are important to prevent the spread of COVID-19 and other pandemic/epidemic diseases. Hence, the purpose of this paper is to review the latest developments in nanobiosensors that provide real-time data on the presence of the virus [14,15,16,17,18]. This will provide the necessary early detection of respiratory viruses, especially 2019-nCoV in schools, workspaces, and other crowded, enclosed spaces. Many researchers in engineering, physics, chemistry, biology, and medical fields focus on the development of smaller, more sensitive, and more selective nanobiosensors, which will offer more precise and targeted detection of the virus, as well as offer environmental monitoring.

Biosensors commonly comprise a biological recognition molecule immobilized onto the surface of a signal transducer and can be used for analysis, diagnosis, safety, protection, and testing of larger populations [14,15,16,17,18,19].

Currently, the most used diagnosis tests are chest computed tomography (CT), reverse transcriptase-polymerase chain reaction (RT-PCN) for RNA detection, lateral flow assays (LFA), full automatic chemiluminescence method, and enzyme-linked immunosorbent assay (ELISA) for the determination of antibodies [20]. Many conventional detection methods of respiratory viruses, such as RT-PCN are time-consuming, expensive, are not always determinable or reproducible, and require trained staff and other specialized facilities. As a result, new techniques made available by nanobiosensors offer simple instrumentation and rapid virus detection, most of them in real-time and at low prices, and are of great interest (in context of the current pandemic) [21].

For rapid diagnosis, early stage disease detection, and identification of infectious pathogens causing the pandemic, nanotechnology can be used as a tool to advance development in medical and environmental applications [22], especially in increasing the efficiency and quality of the detection process by using nanobiosensors [4,23,24]. Moreover, nanotechnology is inspired by virology to develop novel delivery tools to eradicate the viruses that caused epidemics and pandemics, making the development of devices in a scale, ranging from one to a few hundred nanometers, possible [25,26]. At this scale, novel nanostructures [6,27,28,29] and nanosensors exhibit properties and performances unseen at the macroscopic level, especially for detecting and sensing events at a nanoscale level [25,26,30].

Numerous research reports [1,3,5,12] describing the importance of nanotechnology solutions to assess the effects of the COVID-19 pandemic from the detection, protection, and medication perspectives, can be found in open source literature [31,32,33,34]. The development of nanobiosensors, new nanomaterials, and nanofabrication techniques has encouraged researchers in biosensing to search for means to increase the surface area of the biosensing structures, leading to higher sensitivity and shorter detection time than conventional ones. One possibility is to use nanomaterials as indicators for sensing applications [35]. These sensors are able to recognize the analyte of interest, such as protein [36], nucleic acid [37], carcinogens [38], bacteria [39], viruses [40], antibodies, antigens, and other biological components [41] on the surface of a signal transducer [29,42,43,44]. Hence, the biosensing interface layout is important in verifying the efficiency and performance of the nanobiosensor [44,45,46].

Nowadays, researchers improve the specificity and sensitivity of the sensors by utilizing nanocomposites and exploring the chemistry of the surface [47,48], such as nanofilm [49], nanoparticles [50], quantum dots [51,52,53,54], nanowires [55,56], nanorods [27], nanopillars [57], or carbon nanostructures [58]. In addition, the fabrication of three-dimensional (3D) microstructures, nanostructures, and pillars can have a significant impact on controlling and increasing detection mechanisms [59]. With several combinations, the detection performance of the nanobiosensors can be enhanced. These properties make them suitable for medical and environmental applications due to their instant responses and detection. Because of the urgent circumstances, detection of respiratory viruses, including the new coronavirus SARS-CoV-2 (2019-nCoV), is incredibly important in medical, environmental, and social sectors for major protection applications. One of the important advantages of nanobiosensors is the great capability to detect bacteria and viruses at very low concentrations [29]. Consequently, early detection will assist and alert clinicians before the onset of symptoms, with minimum viral effects.

In this paper, the typology of respiratory viruses, including coronaviruses and related nanobiosensors, are reviewed based on the latest research studies, to assess technology utilization in the diagnosis and detection of respiratory viruses, rapidly and accurately.

## 2. Origins, Classifications, and Structures of Human Coronaviruses

Viruses are parasites and have the capability to replicate rapidly when they find a host. However, cells have developed protection mechanisms to recognize and hinder viral replications that could destroy the immune system. Historically, viruses have shown huge epidemiological and pandemic potency: severe acute respiratory syndrome (SARS) in 2002, pandemic swine flu in 2009, MERS in 2012 and, at an unprecedented scale, in 2019, COVID-19. Human coronavirus (HCoV), such as HCoV-NL63, HCoV-HKU1 [60], HCoV-OC43 [8], SARS Coronavirus-1 and 2 (SARS-CoV and SARS-CoV-2) [8,16,61], and MERS coronavirus (MERS-CoV) [62] are human respiratory infections caused by coronaviruses.

**Figure 1:**
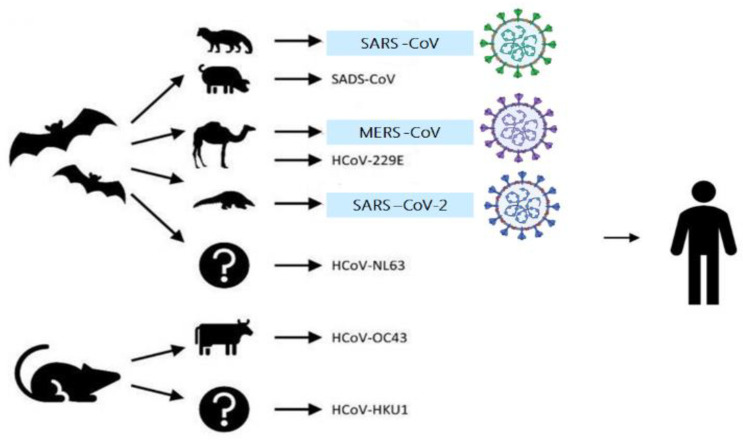
The animal origins of human coronaviruses (severe acute respiratory syndrome coronavirus (SARS-CoV), severe acute respiratory syndrome coronavirus 2 (SARS-CoV-2), and Middle East respiratory syndrome coronavirus (MERS-CoV)). Adapted from Rabi et al. [8], licensed CC BY 4.0.

The primary source of human coronaviruses (HCoV), such as SARS-CoV, SARS-CoV-2, and MERS-CoV are represented by mammals, such as bats, rodents, or mice [8,61], transmitted through other animals or birds as intermediate sources who live in close proximity to people. The animal origins of human coronaviruses (SARS-CoV, SARS-CoV-2, and MERS-CoV) are presented in Figure 1.

HCoV, as well as other types of viruses, including 229E, OC43, and NL63, generally infect the human upper respiratory tract [61], as presented in Table 1.

**Figure 2:**
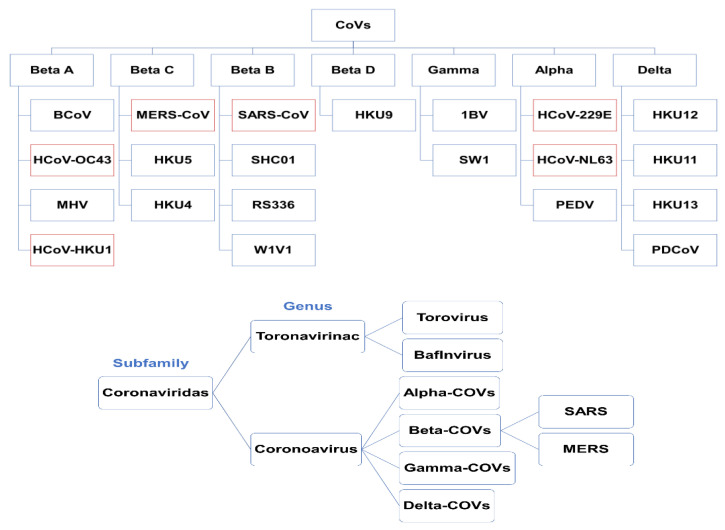
Classification of human coronaviruses (HCoVs) from all four gen-groups. HCoVs are marked with red outlines. Adapted from Monajjemi et al. [61], licensed CC BY 4.0.

According to its genomic structure, coronavirus is classified into four broad categories: alpha, beta, gamma, and delta [8]. Schematic trees of human coronaviruses (HCoVs) created from all four gen-groups are illustrated in Figure 2. Alpha and beta coronaviruses infect only mammals, usually causing respiratory symptoms in humans and gastroenteritis in other animals [8,63,66].

Alpha-CoVs includes human coronaviruses, such as HCoV-229E and HCoV-NL63, and bat coronaviruses. Different types of beta-CoV may infect a wide range of mammals, such as mice and humans. These types include SARS-CoV, HCoV-OC43, HCoV-HKU1, and MERS-CoV, murine coronavirus (MHV), and bovine coronavirus (B-CoV). Gamma-CoVs are specific to birds, except for beluga whale coronavirus. Delta-CoVs were discovered in 2012 with several subunits (HKU11, HKU12, and HKU13) [61]. Coronaviruses are positive-sense RNA viruses that belong to the Coronaviridae family of the nidovirales order, and the coronviridae subfamily [8,61,66,67].

**Figure 3:**
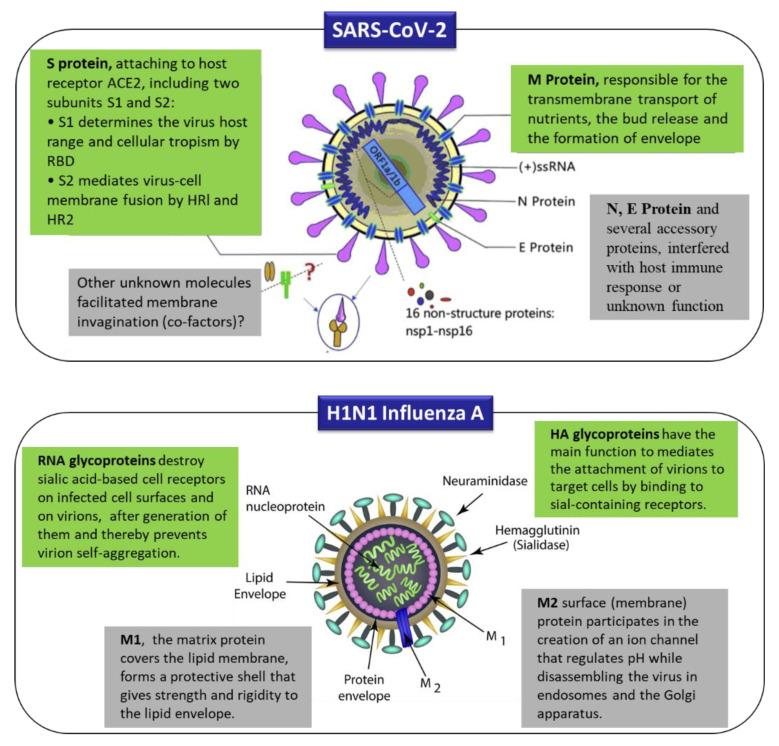
Schematic diagrams of the structural proteins components of SARS-CoV-2 (top), including spike (S) glycoprotein, small envelope (E), matrix (M) protein, and nucleocapsid (N) protein, as well as several accessory proteins [20] (licensed CC BY 4.0), and H1N1 influenza virus structure (bottom), including spikes made up of proteins, such as hemagglutinin (HA) and neuraminidase proteins (NA), matrix protein (M1), and ion channel or M2 protein. Adapted from Besednova et al. [70], licensed CC BY 4.0.

The structural protein components of SARS-CoV-2 are spike (S) glycoprotein, small envelope (E), matrix (M) protein, nucleocapsid (N) protein, and several other accessory proteins. The spike (S) glycoprotein is critical for the host cell adhesion. The SARS-CoV-2 spike (S) protein binds with high affinity of the host cell receptor of SARS-CoV, the host cell receptor known as angiotensin-converting enzyme 2 (ACE 2) [9,68,69]. The M and E proteins are essential for virus assembly. The M protein is responsible for the transmembrane transport of nutrients and the formation of envelope, and the N and E proteins, and other several accessory proteins, obstruct the host immune response or have other unknown functions. S and N proteins are among the most valuable antigen biomarkers for diagnosing coronavirus disease 2019, similar to many detection methods for diagnosing SARS based on S and N proteins [20]. The schematic diagram of structural protein components of SARS-CoV-2 and H1N1 influenza is presented in Figure 3.

These proteins can be essential bioindicators that can be used to enhance the sensitivity and selectivity of nanobiosensors.

To detect the COVID-19 causative virus, the coronavirus shows spike protein immunogens [71]. The generation of immunoglobulins by the immune system increases the detection mechanism. Accordingly, immunoglobulins are important for the detection and possible treatment of COVID-19 [72]. The mechanism of coronavirus infection and replication cycle is very important for choosing the right detection method applications and laboratory tests.

**Figure 4:**
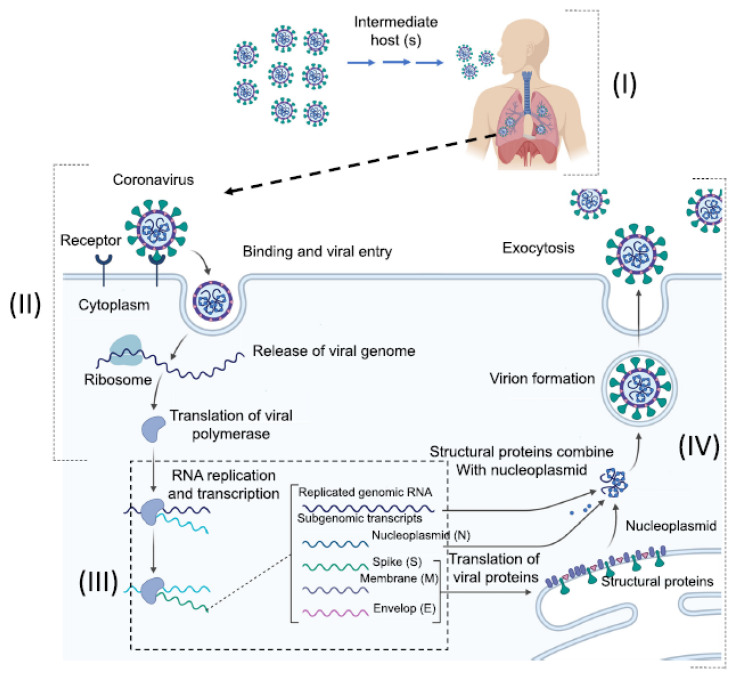
Illustration of the mechanism by which coronavirus infection occurs and its mode of replication. Reprinted from Acter et al. [73]. Copyright 2020 with permission from Elsevier.

According to Acter et al. [73], the mechanism by which coronavirus infection occurs, and its mode of replication/transcription (Figure 4), are as follows: (i) people with SARS-CoV-2 virus through the environment by an intermediate host; (ii) in the first phase, coronavirus connects to the alveolar cells in the lungs through the ACE 2 enzyme using spike (a special surface of glycoprotein), and in the second phase, it enters in the host cell; then, the virus detaches and the RNA genome penetrates the cell cytoplasm, attaches to the ribosomes of the host cell and undergoes translation of viral polymerase; (iii) RNA replication and transcription: nonstructural proteins combine and form RdRp, which represents a multi-protein replicase-transcriptase complex (RTC), RNA polymerase RdRp, and finally RdRp synthesizes positive-sense genomic RNA as descending viruses by replication and subgenomic transcripts; (iv) the host’s ribosomes translate the messenger RNA into the structural proteins, the viral structural proteins (S, E, and M) combine with nucleoplasmin by protein–protein interactions, resulting in viral formation, which finally is released from the host cell.

Contagious respiratory diseases that have caused pandemics, such as flu (e.g., swine flu in 2009), SARS 2002, MERS 2012, and COVID-19 have similar symptoms, although these diseases are caused by different viruses (Table 1) and have different infection mechanisms. For comparison, the mechanism by which infection with the influenza virus occurs and replicates has the following main stages: (i) binding influenza virus with cell receptors (adsorption); (ii) penetration of the influenza virus into the cell (endocytosis); (iii) fusion with endosome (nuclear entry); (iv) transcription, transition, and replication of viral genome segments; and (v) the release of free nucleocapsid in the cytoplasm (fusion sites) [70].

Based on similar aforementioned symptoms (fever, cough, shortness of breath, or difficulty breathing) of contagious respiratory diseases, it is difficult to differentiate the respiratory viruses that caused these epidemic/pandemic diseases. As a result, it is necessary to test the effects of these diseases by different methods [6,60,74,75,76,77,78,79], firstly to confirm the diagnosis, and secondly to detect the viral nucleic acid, specific viral proteins,, or virions of human coronaviruses (HCoV), the newest and most deadly one, the 2019-nCoV (SARS-CoV-2) viruses.

## 3. Significance of Biological Receptors

Nanobiosensors offer several benefits that make detection highly effective, including achievable process, unique performance, high sensitivity, fast response, miniaturization, portability, and accuracy [80]. Nanobiosensors are devices in which the transducer [81] is modified to capture the target element, to convert the biological response into electrical signals, and to quickly detect it with high accuracy [82].

The biological responses can be measured by the determination of the suitable bioreceptors, such as nucleic acids, antigens, DNA probe, peptide, whole cell, micro-organism, and tissue [21,83]. These receptors are easily recognizable, highly sensitive, and able to detect specific bioanalyte. Different types of bioreceptors have been explored to detect the viruses, such as nucleic acids (NA), immunoaffinity and protein in several types of nanobiosensors based on electrochemical, impedance, quartz crystal microbalance, and optical and surface plasmon resonance [25].

**Figure 5:**
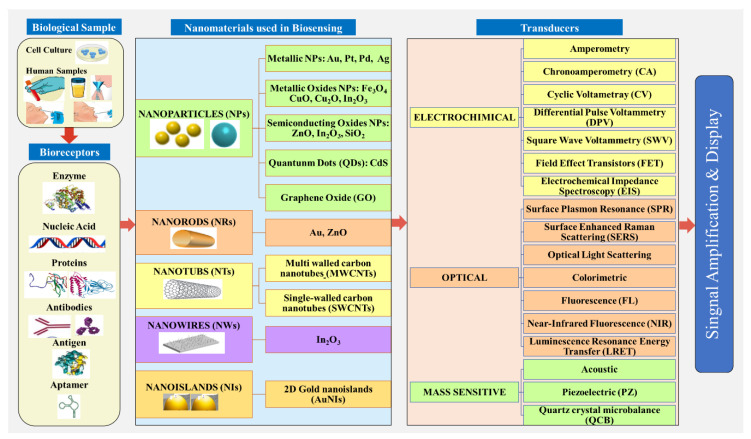
The schematic diagram of different analytes, bioreceptors for biorecognition elements, transducers with integrated nanostructures as parts of a typical nanobiosensor design for respiratory viruses.

The target molecules in the case of respiratory viruses that cause pandemics are viral proteins (antigen, antibody), viral particles, viral nucleic acid, etc. [31,84,85]. The principal structural proteins in novel coronavirus 2019-nCoV that may be suitable targets for viral detection are spike (S) protein, membrane (M) protein, envelope (E) protein, and nucleocapsid (N) protein, and, in the case of other contagious respiratory diseases that have caused pandemics, such as swine flu, the targets are influenza virus M1 protein [86,87], or hemagglutinin (HA) and RNA glycoproteins and neuraminidase (NA) [70], as illustrated in Figure 3. The target molecule attaches to the bioreceptor [88] to detect a biological molecule by a particular reaction. Then, the transducer with integrated nanostructures converts the detection into an electrical signal determined by the detector [89]. The schematic diagram of different analytes, bioreceptors for biorecognition elements, transducers with integrated nanomaterials used for biosensing, as parts of a typical nanobiosensor for respiratory viruses, are presented in Figure 5.

Moreover, an overview of different biological samples, bioreceptors for biorecognition elements, and transducers with integrated nanostructures that are used in literature as parts of typical nanobiosensors for respiratory viruses are presented in Figure 5.

**Figure 6:**
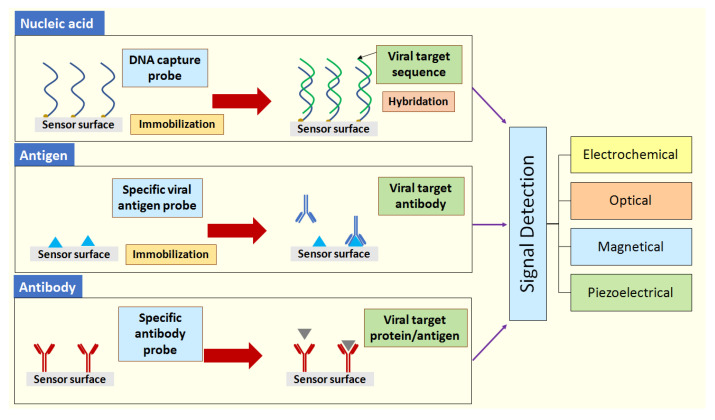
Schematic representation of different biosensors classifications for the detection of SARS and MERS coronaviruses.

Nanobiosensors used for the detection of SARS or MERS coronaviruses, can be classified based on the biological molecule of viral target (nucleic acids, antigens, or antibodies) into nucleic acid-based biosensor, antigen-based biosensor, and antibody-based biosensor (Figure 6).

Biomarkers play a critical role in the fabrication of nanobiosensors for immediate detection of human coronaviruses, including 2019-nCoV. Layqah et al. [62] used spike protein S1 as a biomarker for the detection of highly pathogenic Middle East respiratory syndrome coronavirus (MERS-CoV). As a detection method, they used an efficient, single-step, sensitive, and accurate square wave voltammetry (SWV) with a limit of detection of 0.4 pg/mL. To prevent pandemics, the analysis of viral affinity for human or avian sialoglycan with high sensitivity at high speed is crucial.

With the use of nanomaterials and nanotechnology, ultrasensitive biosensors for the detection of antigens are developed. In various studies of patients with COVID-19, biomarkers have shown significant response by using testing samples from healthy and infected patients. Table 2 includes the results of some studied biomarkers in medical clinics.

The clinical results included in Table 2 were obtained after 452 patients diagnosed with COVID-19 [90]. A major issue needs to be taken into consideration when using biomarkers for COVID-19. For instance, these biomarkers must be user-friendly to ensure the safety of the professional who is testing them from transmitting the virus.

**Figure 7:**
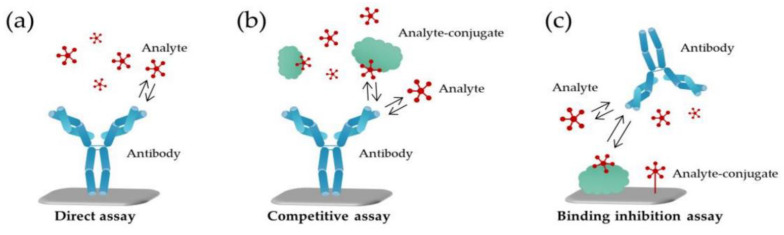
Schematic representation of different assay formats in the detection of small molecules. (**a**) In a direct assay, target analyte attaches to the antibody (recognition element) immobilized on the sensor surface; (**b**) in a competitive assay, the analyte competes with its conjugate to attach to the antibody; (**c**) in a binding inhibition assay, the analyte conjugate is the one immobilized on the sensing surface [94]. (Licensed CC BY 4.0).

The mechanism of surface detection can be varied based on the interaction process between the bioreceptor and the analyte of interest. Figure 7 shows different assay formats used for the detection of small biomolecules [94].

Label-free nanobiosensors are based on the direct interaction with the target analyte, where the detection is achieved with the biological recognition element immobilized on the surface of the nanobiosensor (Figure 7a). In case of competitive (Figure 7b) and binding inhibition assay (Figure 7c), an intermediary part between the analyte and the biological recognition element immobilized on the surface of the nanobiosensor is required to increase the detection ability. In case of the competitive assay, the sensing area is coated with the recognition element, whereas the analyte and its conjugated equivalent compete to attach into a finite number of binding sites on the sensing surface. However, in case of inhibition detection assay, there is a reverse process, i.e., the analyte conjugate is immobilized on the sensing surface, while the recognition element is connected to solution of the analyte [95]. Consequently, no matter what type of assay format is chosen, it is important to determine the immobilization phase of the exposed sensing area in the structure of the nanobiosensor. Hence, the surface modification and functionalization should be a selective process that supports the binding and interaction of the analyte of interest.

## 4. Nanobiosensors for the Detection of Human Coronavirus (2019-nCoV, SARS/MERS-CoV) and Influenza Viruses

Nanobiosensors are important tools for efficient detection of severe acute respiratory syndrome and other diseases that cause pandemics [96,97,98,99,100,101]. For virus detection, depending on the detection mode and measurable properties, nanosensors can be classified into different classes, such as electrical, chemical, electrochemical, optical, piezoelectric [102], thermal [103,104], magnetic [105], and biological detection [57]. In the following sections, a brief review of the nanobiosensor detection mechanisms of epidemic/pandemic respiratory viruses, such as avian influenza virus (AIV) subtype H5N1 (epidemic in 2004) or H7N9 (epidemic in 2013) specific to avian/bird flu, human influenza A virus subtype H1N1, specific to swine flu (pandemic in 2009), seasonal H3N2 flu, and coronaviruses, such as SARS (epidemic in 2002), MERS (epidemic in 2012), and the COVID-19 causative virus (2019-nCoV), and their parameters, are presented.

### 4.1. Electrochemical Nanobiosensors

Electrochemical nanobiosensors have been applied in several fields [25,26,29,57,106,107]. An electrochemical nanobiosensor is a molecular sensing device that couples a biological recognition event with an electrode transducer to produce a useful electrical signal [108]. Electrochemical nanosensors contain electrodes, for which the semiconductors properties, dielectric properties, and charge distribution are important factors.

Surface modification of the nanostructures or nanomaterials is required to modify the functional layer in order to obtain definite selectivity for a given sensing surface. The various studies have been performed to enhance the performance of nanobiosensors by incorporating nanomaterials in the surface to increase the volume-to-surface area ratio and improve the selectivity of the surface. As shown in Figure 5, nanomaterials have a great impact when used in biosensing applications. Most of the nanobiosensors used for detection of pathogen and viruses are based on electrochemical transducers, i.e., amperometric, potentiometric, or impedance transducers [109]. The mechanism of the nanobiosensor is based on the change in the electrical conductance or resistance through the nanostructures or nanomaterials, when the target element attaches to the electrode surface. When the electron consumption or production occurs in a biological reaction on the electrode surface, an electrochemical signal is generated. The selectivity leads to the ability to measure only one chemical in the presence of many other chemicals in real time with high detection accuracy. The general mechanism of sensing is based on the chemisorption of molecules that induce a change in conductance due to chemical sensitization.

Bioreceptors must be integrated effectively into the right transducer to detect the presence of any virus within the target sample. For the detection of different types of coronaviruses, most researchers used electrochemical detection methods, such as field effect transistor (FET) [69,110,111], bioelectric recognition assay (BERA) [111], electrochemical impedance spectroscopy (EIS) [78,112,113,114,115,116,117], amperometry [27,118,119], cyclic voltammetry [53,62,87,108,120,121,122], or conductometry [55,123]. Influenza is an acute infectious disease caused by a coated ribonucleic acid (RNA), which contains viruses belonging to the Orthomyxoviridae family [80]. Influenza has four types A, B, C, and D, which differ in the structure of the virus (ion channel, matrix, and membrane protein). Individual differences could help detect individual subtypes of influenza virus [80,81,82]. Influenza A viruses are divided into subtypes on the basis of two proteins on the surface of the virus: hemagglutinin (HA) and neuraminidase (NA). For example, the most studied is the influenza A virus subtype H1N1 (A/H1N1), which was responsible for the 2009 swine flu pandemic, as well as the 1918 flu pandemic, known as the Spanish pandemic flu, which caused millions of deaths, are still a threat to people in many countries.

Avian influenza virus (AIV) H5N1, H7N7, and H7N9, which are subtypes of influenza A virus, were studied by many researchers [115,116,117] through EIS, amperometry [27], cyclic voltammetry [120], etc. The biological response was transferred to the detector via a transducer integrated or functionalized with nanostructures, using different types of nanomaterials, such as magnetic iron oxide nanobeads [115]. A second category of respiratory viruses that caused epidemics or pandemics are coronaviruses, including SARS-CoV, MERS-CoV, and the novel coronavirus 2019-nCoV, which is the most deadly one. For these aforementioned reasons, many researchers studied respiratory viruses specific to seasonal influenza through different types of electrochemical methods, along with the biosensors designed for respiratory coronavirus detection that causes COVID-19 disease. These studies are summarized in Table 3, which shows a comparison of electrochemical detection methods of influenza viruses, human coronaviruses (including recently developed COVID-19 causative virus) and their parameters.

Table 3 shows that impedance biosensors are the most sensitive compared to other types of biosensors, such as optical or mass sensitive, and are ideal for detecting specific proteins or DNA strains, or for monitoring the environment [86,142]. Electrochemical impedance spectroscopy (EIS) is less destructive for measuring the interactions between biological samples, in comparison with other electrochemical methods, such as differential pulse voltammetry (DPV), cyclic voltammetry (CV), etc. [143].

Many research groups studied the M1 protein of influenza A virus using covalently bound antibodies and a nano-scale boron-doped diamond surface sensor [86,113]. Nidzworski et al. [113] developed a rapid-response, ultrasensitive biosensor for influenza virus detection using an antibody modified boron-doped diamond, and obtained a limit of detection of 1 fg/mLa and a linear range of 1–100 fg/mL. Similarly, Siuzdak et al. [86] obtained LOD: 5 × 10^−14^ g/mL. Both groups of researchers used saliva as biological samples from the tested subjects, which is easy to collect and analyze by EIS-based biosensors.

To analyze the saliva samples and detect the nCovid-19 spike antigen (nCovid-19Ag), Mahari et al. [141] used an in-house built printed circuit board for fast detection of nCovid-19 antigen (nCovid-19Ag). The sensor was manufactured using a fluorine-doped tin oxide (FTO) electrode with gold nanoparticles (AuNPs) and immobilized with nCovid-19 (nCovid-19Ab) monoclonal antibody to measure the change in electrical conductivity. They used different detection methods, such as cyclic voltammetry (CV) and differential pulse voltammetry (DPV), and obtained very good sensitivity, as follows: the limit of detection was 10 fM of nCovid-19 Ag and the linear range was 1 fM to 1 μM in standard buffer [141]. Veerapandian and co-workers [121] developed an electrochemical biosensor based on chronoamperometry and differential pulse voltammetry (DPV). Graphene oxide nanosheets and immobilized H5N1 and H1N1 antibodies were used to examine the detection and response of the nanobiosensor. The limit of detection was measured to be 9.4 pM for H1N1 and 8.3 for H5N1. The use of antibodies revealed an important interaction with the corresponding antigen. The biosensor showed an increase in all necessary parameters, such as sensitivity, speed, and simplicity at a lower cost.

Various pathogenic viruses, including SARS-CoV-2 spike proteins can be used as reliable markers for the presence of the infection and virus replication. For direct and ultra-rapid detection of SARS-CoV-2 (3 min and 1 fg/mL concentration) S1 spike protein antigen from green monkey kidney cell culture samples [4], the bioelectric recognition assay (BERA) was used. Over the years, various electrochemical nanobiosensors have been used to detect human influenza A virus (such as H1N1) using electrochemical impedance spectroscopy (EIS) [78], chronoamperometry [27,118,126], voltammetry [135], or conductometry [55].

#### 4.1.1. FET-Based Electrochemical Nanobiosensor

Field-effect transistor (FET)-based biosensors have several advantageous and properties compared to other techniques. These biosensors can be highly sensitive and provide instant measurements with very low concentration of the bioanalytes [144,145]. FET-based biosensors can be utilized in a number of applications, particularly in medical care, point-of-care testing, and diagnosis [146,147,148].

Graphene is an advanced nanomaterial that consists of a two-dimensional layer of carbon atoms [149]. This material can be used as an active sensing surface due to its excellent electrical conductivity, high carrier mobility, simplicity of surface functionalization, and large surface area [150]. Therefore, to optimize the sensitivity of detection, the integration of graphene-based material can be the best candidate for FET biosensors.

To prevent an influenza pandemic, it is necessary to differentiate with high accuracy, speed and sensitivity the viruses of pandemic potentials on the basis of the viral affinity for sialoglycan [110]. In this case, and also for highly sensitive detection of biological substances, such as DNA and proteins (like lectin), the graphene-based field effect transistor (G-FET) method was used. For rapid detection, high stability and sensitivity of biological targets, such as the H5N1 influenza virus gene, and the bio-FET method, as a promising platform for label-free detection via a flow-through strategy, was used [111]. For instance, high sensitivity was reported by Ishikawa et al. [45]. Ono et al. [110] and Chan et al. [111] reported that Limit of Detection (LOD) of influenza viruses are in the picomolar range, i.e., 130 pM and 50 pM, respectively. For influenza detection, Ono et al. [110] used a single-layer hexagonal carbon networks (grapheme FET) for selective detection of lectins, while Chan et al. [111] used reduced graphene oxide (rGO) films on Si/SiO_2_ substrate for increasing the sensitivity of Bio-FET sensor for H5N1 influenza virus gene detection in a flowing environment.

**Figure 8:**
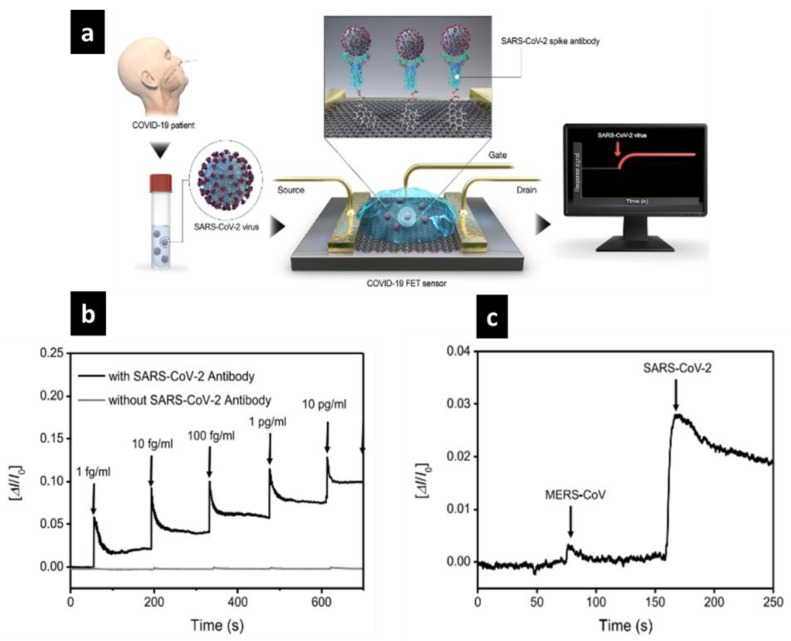
(**a**) Schematic illustration of graphene-based field effect transistor (FET) biosensor mechanism and detection, where SARS-CoV-2 (spheres) binds to antibodies (Y-shapes). (**b**) Real-time response of COVID-19 FET toward SARS-CoV-2 spike protein. (**c**) Bionanosensor selectivity response toward two different proteins: SARS-CoV-2 and MERS-CoV. Reprinted from Seo et al. [69]. Copyright 2020 American Chemical Society.

Seo et al. [69] developed a FET biosensor for the COVID-19 causative virus, which contains a sheet of graphene-based material functionalized by attaching antibodies alongside the SARS-CoV-2 spike antibody (Figure 8a).

The SARS-CoV-2 spike antibody is used as a bioreceptor because it is a transmembrane protein, immunogenic, and has the selectivity to detect SARS-CoV-2. If the SARS-CoV-2 antigen protein is exposed, an electrical response is obtained because of its binding to the antibody. The results of the electrical characterization of this biosensor showed an effective response to the COVID-19 detection by integrating a graphene-based FET biosensor.

Figure 8c shows the real-time detection of 1 fg/mL of SARS-CoV-2 spike protein compared to that without SARS-CoV-2 spike protein which did not reveal any response as the concentration of the samples was changed. In addition, Figure 8 reveals the selectivity and sensitivity of the biosensor to differentiate the SARS-CoV-2 antigen protein from others such as MERS-CoV, leading to the surface ability to bind particularly with the selected SARS-CoV-2 antigen. The results of this biosensor showed a remarkable detection of the virus, which could be used in the future as it is, or modified for testing other diseases.

#### 4.1.2. Cell-Based Electrochemical Nanobiosensor

The estimation of the number of infected patients in real-time is one of the major challenges in the management of the recent COVID-19 disease. A novel biosensor for faster detection of the SARS-CoV-2 S1 spike protein in 3 min and ultra-sensitive surface, with a detection limit of 1 fg/mL, has been recently developed by Mavrikou et al. [4].

**Figure 9:**
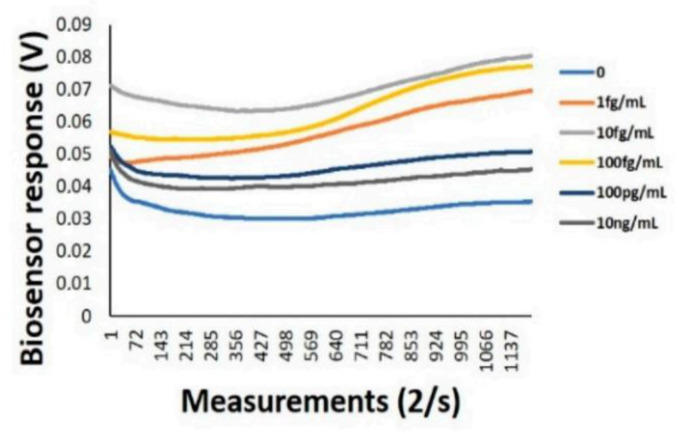
Biosensor response given by the variation of voltage in time for different concentrations of biomolecules [4] (licensed CC BY 4.0 from *Sensors,* 2020).

Membrane engineering, cell-based assay concept for the determination of biomolecules has been utilized to enhance the attachment of a particular protein to cellular components that were obtained by electro-inserting spike S1 antibody. It has been found that biomolecules binding to the electro-inserted antibodies provided a successful change in electrical measurements of the engineered cell membrane [151,152]. The results have shown significant difference in the properties of electrical behavior, as shown in Figure 9. The developed biosensor has different advantages for the clinical testing, monitoring, and managing the virus. For example, this biosensor has a portable read-out device, easy to handle, and mass screening of SARS-CoV-2 surface antigens.

### 4.2. Optical Nanobiosensors

In recent decades, new photonic devices have looked promising for wide-range applications in the field of nanobiosensor technology for food safety, homeland security, biology, environmental monitoring, and medicine [153,154,155]. Different parameters can be used in detection, such as energy, polarization, absorption, fluorescence, light scattering, amplitude, decay time, and/or phase [156].

The surface plasmon resonance (SPR) transduction of the optical nanosensors can determine the variation in reflective index of the transducer as the target analyte interacts with the biorecognition element on the surface of the sensor [157].

Fluorescence involves the exposure to an external light source to excite the electron transitions in the biomolecules, which then generate luminescence. Hence, this type of optical biosensor requires the integration of fluorochrome molecules to generate light during the interaction with the immobilized biorecognition element [158].

Optical fibers in biosensing applications have received special attention due to their high sensitivity, high performance, and fast response [159,160]. Optical fibers are commonly integrated with surface plasmon resonance or fluorescence in various applications to monitor the changes of the optical properties, such as the wavelengths wave propagation, time, intensity, or polarity of the light to detect the analytes of interest [161]. These properties can be measured to detect the analyte concentration. For example, an amplitude is the most important parameter that is correlated with the concentration of the analyte [162]. Optical fiber biosensors have been widely used for the detection of pathogen, virus and bacteria [161,163,164]. Table 4 shows a comparison of optical detection method of influenza viruses and other coronavirus, including recently developed COVID-19 causative virus nanobiosensors and their properties.

The nanobiosensors based on surface plasmon resonance [46,170,192,193], and fluorescence principle are the most common sensors because they demonstrated a high potential for optical based detection of viruses, including the SARS-CoV-2 coronavirus [194]. The SPR sensor system is extremely sensitive, has a rapid response time, is label-free and has real-time detection of binding events between biomolecules and the surface, and has the advantage for detecting the changes at nanoscale interface with high accuracy.

For instance, surface plasmon resonance-based biosensor was used by Miodek et al. [87] to immobilize specific anti-PB1-F2 antibody on the surface of the modified gold microelectrode. This type of biosensor has the ability to perform direct and quantitative detection of PB1-F2 protein of influenza A virus in infected cells, leading to the exploration of the capability of the biosensor detection for other viral proteins in infected cells, tissues or biological samples. The successful detection of SARS-CoV antigen via SPR analytical systems with reference (high-throughput, multianalyte imaging SPR analytical system) by directly immobilizing antibodies was achieved by Dafu et al. [195]. With the improved SPR reference system (improved system by eliminating non-specific disturbances and avoiding the inference of refractivity of different solutions at different temperatures), samples collected from patients can be analyzed without pretreatment.

Among the different biosensing techniques, localized surface plasmon resonance (LSPR) biosensing systems are applicable to many pathogenic and viral agents, such as different types of influenza viruses, SARS-CoV, MERS-CoV, and SARS-CoV-2 viruses. Qiu et al. [1] developed a highly sensitive, fast, and reliable dual-functionalized LSPR biosensor for the detection of selected sequences from severe acute respiratory syndrome coronavirus 2 through nucleic acid hybridization.

The concept of double functionality of the plasmonic biosensing was integrated with the plasmonic photothermal (PPT) effect and the LSPR sensing transduction on a single cost-effective gold nanoislands (AuNI) chip. Ultra-sensitive detection was successfully developed by Park et al. [77] with SPR imaging and localized SPR-based optical biosensor for label-free monitoring target molecules of influenza B virus (with a limit of detection up to the 1 pg/mL).

Fluorescence is frequently used to detect viruses (viral protein) and monitor viral infection of cells [196]. The fluorescence-based detection system requires the labeling of the process for obtaining the fluorescence signal, on the principle of the on/off signal between the binding of the target to the fluorescent probe (e.g., fluorescent nanoparticles/proteins, dye-labeled nucleic acid, etc.). Fluorescent labels in biosensors for pathogen detection offer a user-friendly, fast, efficient, and low-cost biosensing systems for pathogen monitoring [197]. For instance, Nguyen et al. [54], developed a fluorescence biosensor based on CdTe quantum dots, for the specific detection of influenza virus H5N1 type (with 3 ng/μL LOD for H5N1). The biosensor included highly luminescent CdTe/CdS quantum dots, antibody, and chromatophores extracted from particular bacteria. This part of the biosensor was then connected to a peripheral part of the biosensor and also connected to the H5N1 antibody to make it ready for the detection of the H5N1 avian influenza virus. The advantage of this fluorescence biosensor consists in the flexibility of the peripheral part to detect any other types of viruses only by replacing the specific antibody to the determined target virus [54]. Waye et al. [180] used also the fluorescent detection of the 3a Protein (3a gene encodes a non-structural viral protein) of SARS-Coronavirus. The chromatin condensation experiments and DNA fragmentation were performed in vitro.

Chemiluminescence is a luminescence technique similar to florescence used in nanobiosensors, i.e., it detects the light emitted by atoms and molecules when the electron relaxes from the excitation level. Although similar in detection mode, chemiluminescence nanobiosensors diverged from fluorescence at one point in time when advanced chemiluminescence array for magnetic separation were developed. In fluorescence, the light is released when the electrons relaxes from higher energy states, while in chemiluminescence, the light is released when the excited electrons in unstable intermediary states relax to produce the final reaction products. Xi et al. [198] developed a chemiluminescence sensor with a detection limit for HBsAg, a marker of the Hepatitis B virus, as low as 0.05 ng/mL, which is ten times lower than the typical ELISA used in hospitals. More recently, to improve the collection efficiency of chemiluminescent emission induced by samples, Nie et al. [199] designed a chemiluminescent optical fiber sensor by using a concave mirror and a coaxial tubular mirror as its bottom and wall, respectively. The limit of detection was as low as 0.31 pg/mL [199], which is about 2 orders of magnitude lower than that obtained by a normal chemiluminescent optical fiber sensor.

In immunofluorescence detection of viruses, nanobiosensors were developed based on the gold nanoparticles (AuNP)-induced quantum dot (QD) fluorescence signal, with a detection limit of 10 PFU/mL [30]. The H1N1 virus—a virus that belongs to the family of influenza A viruses, such as H3N2—has been responsible for the 2009 flu pandemic and continues to be in the attention of scientists searching for rapid and effective detection in real time. For the rapid diagnosis of influenza virus type A, Park et al. [176] reported a surface-enhanced Raman scattering (SERS)-based lateral flow assay (LFA) kit with a detection limit of 1.9 × 10^4^ PFU/mL, which is approximately one order of magnitude more sensitive than the LOD obtained from the colorimetric LFA kit.

Colorimetric assays are representative tools that basically identify the target molecules in tested specimens through color changes of an indicator (e.g., nanosized metallic particle and dye molecules) and, because of their fascinating optical properties, plasmonic nanostructures have inspired numerous colorimetric detections of biomolecule for a wide range of applications from pharmaceutical to environmental analyses [200].

The colorimetric test offers the advantages of on-site detection of analytes due to its direct reading, convenient operation and minimum instrumentation requirement. This technique allows visual observation of the presence of biomarkers and measures the absorbance of the colored compounds at a specific wavelength. In the actual context of public health emergency caused by the human coronavirus (HCoV), a simple and fast colorimetric assay for detecting infectious disease (with naked eye and without costly equipment) is still imperative.

Thus, Kim et al. [186] developed a label-free colorimetric assay for MERS-CoV based on an extended form of double-stranded DNA (dsDNA) self-assembly shielded gold nanoparticles (AuNPs) under positive electrolyte (e.g., 0.1 M MgCl_2_), for the detection of MERS-CoV. This colorimetric test can detect up to 1 pmol/μL of 30 bp MERS-CoV and can be further adapted for convenient on-site detection of other infectious diseases, especially in resource-limited settings.

#### 4.2.1. Magneto-Optical Nanobiosensors

Magneto-optic (MO) nanobiosensors present a great interest in the development of ultra-sensitive biosensing application due to their combined magnetic properties and surface plasmonic enhancement that is associated with metal nanoparticles [201,202,203]. MO sensing platforms have exceptional parameters, including label-free biosensing, fast response, and ultra-sensitive detection.

Novel applications of MO nanobiosensors have been successfully reported in the medical field including hyperthermia treatment, magnetic actuation, targeted drug delivery, and the use of magnetic particles [204].

Magnetic nanosensors are obtained by using magnetic beads coated with a ligand, which can be detected by a magnetic field [29]. The SPR transducers in biosensing are highly influenced by the change in the reflective index associated with the binding or the reaction of biomolecules and bioreceptors at the metal surface. The presence of ferromagnetic metals enhance the surface plasmons resonance [205,206,207,208]. Under the applied magnetic field, the ferromagnetic surface can play a role in tuning the SPRs. The properties of MO nanobiosensors are not used only to improve the SPRs, but also to optimize and increase the sensitivity of the sensors. Table 5 presents an assessment of magneto-optical detection methods and their different parameters for the current developed influenza and coronavirus nanobiosensors.

Rapid and ultrasensitive detection (5 × 10^−12^ g/mL, by human eyes and 44.2 × 10^−15^ g/mL, by a microplate reader) of influenza virus type A was determined by Oh et al. [210]. The linear range was in this case from 5 × 10^−15^ to 5 × 10^−6^ g/mL. They used silica-shelled magnetic nanobeads (MagNBs) and gold nanoparticles with a new platform-based ELISA technology. The new MagLISA detection platform has many advantages, such as advanced sample separation, sensitive reading and anti-interference ability that can reduce the spread of influenza virus and provide immediate clinical treatment.

For the detection of SARS-CoV-2 RdRp coding sequences, Tian et al. [14] used optomagnetic sensing and achieved a sub-femtomolar level detection limit of 0.4 fM. Another advantage of the real-time optomagnetic detection of SARS-CoV-2 RdRp coding, compared with previously reported C2CA-based sensors, is to significantly simplify the operation by eliminating the labor-intensive and time-consuming operation steps that require different reaction temperatures.

Developments in chemiluminescence have led to an improvement in the optical signal of a luminophore near the surface of metal nanoparticles [213,214,215,216]. The electromagnetic field of incident light can be enhanced to accelerate the detection and control the energy transfer. This type of optical configuration has emerged as a potential method for the fabrication of various medical applications due to its high sensitivity, simplicity, and low noise [217]. For example, Lee et al. [209] used a magnetofluoro-immunosensing platform for virus detection (i.e., H1N1 Influenza) using Au/Fe_3_O_4_ decorated graphene. The LOD of influenza virus in deionized water was 7.27 fg/mL. The Au/iron oxides decorated graphene was prepared as plasmonic/magnetic graphene, which was used for a target virus separator and a plasmonic substrate. On the other hand, Zhao et al. [212] have used poly(amino ester) with carboxyl groups (PC)-coated magnetic nanoparticle (PCMNPs) to detect SARS-CoV-2 pseudovirus particles. Extracting the SARS-CoV-2 viral RNA, a 10-copy sensitivity and a linear correlation of 10–10^5^ copies of SARS-CoV-2 pseudovirus particles were obtained. The major advantage of this structure is its high performance in the extraction procedure to reduce the detection time in the recent diagnosis of COVID-19 causative virus.

#### 4.2.2. Recently Developed COVID-19 Optical Nanobiosensors

**Figure 10:**
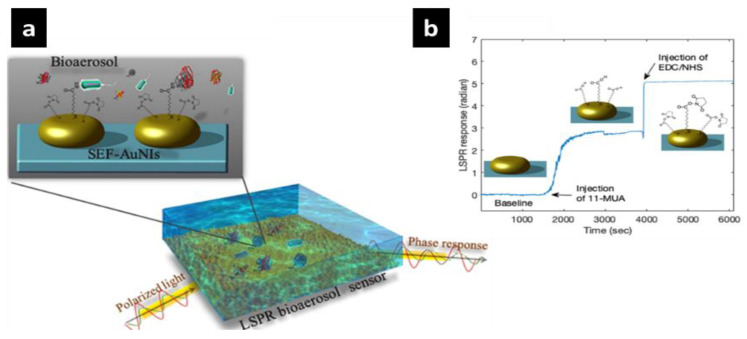
(**a**) Schematic illustration of AuNIs surface functionalization and bioaerosol detection. The surface of the AuINs was functionalized with succinimidyl-ester to detect bioaerosol. (**b**) In situ phase sensing response of surface functionalization, including the anchor 11-mercaptoundecanoic (11-MUA) and activator EDC/NHS. Reprinted Qiu et al. [219]. Copyright 2020 American Chemical Society.

Researchers from Empa and ETH Zurich (Zürich, Switzerland) have developed a successful optical sensor to detect COVID-19 virus [218]. This could be utilized to measure the presence of the virus in the surroundings. The sensor would be considered an alternative method to quantify the virus concentration in the air and in real time, particularly in crowded and busy places. Wang et al. [218] have worked on analyzing and minimizing the presence of airborne pollutants such as aerosols and artificially produced nanoparticles. To enhance the detection in a safe, reliable and more sensitive technique, researchers have fabricated the optical nanobiosensor based on localized surface plasmon resonance (LSPR) phenomena. A LSPR biosensor consists of the succinimidyl ester group that was functionalized on the surface and attached to two-dimensional (2D) gold nanoislands with an average size of 40.2 nm, as active sensing surface on a glass substrate (Figure 10a).

As the light polarized the sensing surface, the plasmonic phase varies to offer superior detection sensitivity for bioaerosol concentration [219,220]. The presence of gold nanoparticles on the surface increases the sensitivity as the biomarkers are illuminated. Figure 10b shows the LSPR response of the functionalized surface of the biosensor, which interacts with the sulfhydryl group of 11-mercaptoundecanoic (MUA) to form in situ surface modification.

A mixture of 1-ethyl-3-(dimethyl-aminopropyl) carbodiimide hydrochloride (EDC)/N-hydroxyl succinimide (NHS) (EDC/NHS) was injected to test the phase changes. As a result, the active succinimidyl ester was sensitive and responded to an amino group, forming an amide bond while identifying bioaerosols. To assess the reliability of the novel nanobiosensor detection on the COVID-19 causative virus, several challenges are still required to validate the nanobiosensor sensitivity to the coronavirus.

**Figure 11:**
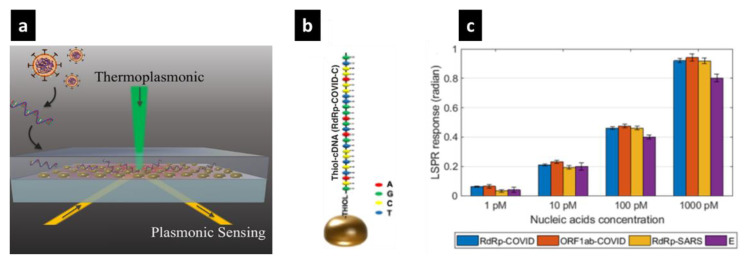
(**a**) Schematic diagram of a plasmonic biosensor. (**b**) Schematic illustration of surface modification of Au nanoisland and the thiol-cDNA ligands. (**c**) Different nucleic acid concentrations measured using the dual-functional LSPR biosensors Reprinted from Qiu et al. [1]. Copyright 2020 American Chemical Society.

Fast and precise detection of the COVID-19 causative virus can greatly promote the management, prevention, and control of an emerging disease [221]. Two different sensing platforms have been developed in a nanobiosensors, including localized surface plasmon resonance, and plasmonic photothermal (PPT) effects Figure 11a [1].

LSPR is a perfect candidate for immediate and label-free detection of micro- and nanoscale biomolecules [222,223]. In LSPR, the surface sensitivity is greatly influenced by the local variation in the refractive index and molecular binding [44]. The PPT heat energy effect, known as thermoplasmonic, is confined close to the nanoparticles to enhance the kinetics of hybridization of nucleic acid strands (RdRp-COVID and its cDNA) to prevent the interaction with nonmatching sequences, leading to better detection mechanism [1,224,225,226]. The developed biosensor provides a promising solution for the detection of COVID-19 causative virus. The combination of optical and thermal techniques leads to the excitation of different wavelengths that highly improve sensitivity, reliability, stability, and rapid diagnostic for SARS-CoV-2 virus.

The plasmonic biosensor consisting of two-dimensional gold nanoislands were self-assembled on the glass surface and then functionalized with corresponding bioreceptors. Figure 11b shows an attachment of the thiolcDNA bioreceptor to the Au nanoisland. The accurate surface functionalization is important to functionalize and to improve the sensitivity of the Au nanoislands sensing surface. For better sensing performance, the thermoplasmonic effect is generated on the same Au surface when irradiated at their plasmonic resonance frequency. The localized PPT heat is capable to detect nucleic acids, to elevate the hybridization kinetics and to facilitate the accurate discrimination of two similar strands. The biosensor with double functionalized capability of sensing model exhibits a high sensitivity toward the selective hybridization recognition of SARS-CoV-2 sequences with a minimum detection limit to the concentration of 0.22 pM, and allows precise detection in a multigene combination. Figure 11c presents the result of LSPR sensing detection with the in situ PPT improvement. This work highlights the importance of plasmonic bionanosensor and its effectivity to the detection of the presence of nucleic acid for various diseases.

### 4.3. Piezoelectric Nanobiosensors

Piezoelectric quartz crystal microbalance nanobiosensors have gained considerable attention in biological and chemical applications particularly for the detection of influenza viruses due to their simple model, direct recognition, and real-time output [227,228,229]. There are two types of piezoelectric sensors, i.e., bulk wave (BW) and surface acoustic wave (SAW). These biosensors are able to detect the biochemical entities [25,29] and convert the mechanical energy into electricity, which provide the user with a usable energy output in response to a specific measurement input. For example, the piezoelectric material generates mechanical resonance of vibrating crystal at its natural frequency. This frequency is influenced by the external electrical signal. As the analyte of interested is exposed to the sensing material, a reaction will eventually occur and produce a shift in the frequency that causes a change in the electrical measurements. Therefore, the detection can be obtained by utilizing microscaled or nanoscaled sensors.

The probe is excited mechanically to resonate and measure the change in mass and viscoelasticity on the surface by tracing the frequency and modifying a quartz crystal resonator [230]. The benefit of this technique is the capability to detect molecules without labeling. However, associated challenges include the detection mechanism complexity and less precise measurements [231]. These biosensors have been used in a wide range of biological applications to identify the presence of some target biomolecules, such as hormones, bacteria, cells, etc. [232,233]. To detect avian influenza virus (H5N1), Wang et al. [227] fabricated a quartz crystal microbalance (QCM) sensor using a 3-dimensional (3D) nanowell onto the surface of gold (Au). The sensitivity limit of detection (LOD) of the sensor exhibited 2–4 HAU/50 μL, and a detection time of 10 min, leading to faster detection time compared to other techniques (30 min) [234]. Among the mass detection-based biosensors, the piezoelectric immunosensor was developed for the detection of SARS-associated coronavirus (SARS-CoV) [102]. The mass-based biosensors are summarized in Table 6, including the recent developed COVID-19 causative virus nanobiosensors and their properties.

The integration of quartz crystal microbalance (QCM)-based method for the detection of several respiratory viruses such as Influenza, SARS-CoV and 2019-nCoV from oral swab has been developed [102,238,240,241]. This method can be used for the label-free testing in real-time response with high sensitivity. QCM-based nanobiosensors are the most appropriate sensor for exploring flat surface. For the detection of 2019-nCoV, oral swab samples were collected to measure the response of SARS-CoV-2 spike protein [241]. The detection method of QCM-based sensors is achieved based on the interactions between the spike glycoprotein and the surfaces of the sensor, which was able to detect the adsorbed spike proteins and, hence, the sensitivity was high in the range of ng level.

For SARS-CoV detection, the utilization of piezoelectric immunosensor has been determined to be fast, stable, and effective [102]. Albano et al. [240] has explored the effect of paramagnetic nanoparticles with using a piezoelectric quartz crystal nanobiosensor to detection protein biomarkers at pg/mL level. Fast detection was obtained in a one-minute assay with a detection limit of 3.5 ng/mL, leading to high sensitivity and selectivity nanobiosensors. This kind of nanobiosensor is highly desirable to detect viruses with high reliability, to determine other respiratory viruses.

## 5. Challenges and Opportunities for COVID-19 Causative Virus Nanosensors

Several challenges in the development of novel nanobiosensors still exist and need to be addressed in the research community and manufacturing industry to have consistent and efficient detection devices. In order to develop a novel nanobiosensor that can overcome the current challenges, different factors need to be taken into account in the manufacturing process of sensors, including techniques for modifying the surface chemistry for immobilization, to improve the detection limit and selectivity for better sensing, and to ensure data manipulation and analysis. Many scientists are focused on improving the sensitivity and detection limit of biosensors using metallic and semiconducting oxides [242].

In addition, multi-tasks nanobiosensors are needed to support future needs for immediate detection. Quantitative comparison of the use of multiple sensors on a chip is important for data management. Furthermore, the development potential of portable and wireless nanobiosensors is very good for diverse applications [20].

For the COVID-19 causative virus, different nanobiosensors have been developed for different applications in medical and environmental fields by academic and industrial sectors. However, no one could guarantee whether nanobiosensors have good diagnostic results to be used on the frontline. Even though the concept of the electrostatic approach to capture the virus is interesting, the selectivity of the surface to detect the presence of the virus has not yet been explored. The major challenges in gapping the development of nanobiosensors from the lab to industry are as follows:

(1) Fabrication of simple, easy to manipulate, early diagnosis, on-site, inexpensive, fast detection, and highly sensitive nanobiosensors could have remarkable potential for many applications, including hospitals, clinics, laboratories, schools, shopping malls, airports, and home.

(2) Increase the accuracy of the diagnosis in the fabrication of multitask nanobiosensors is essential for fast detection.

(3) Improve the reliability and reproducibility of nanobiosensors, it is necessary to build and use machine learning-based programs for the signal process, and to obtain, directly, correct and safe readings of the results.

## 6. Conclusions

Rapid spread of viruses can be prevented if the virus causing the epidemic is identified early. The COVID-19 causative virus spread across the world and became a critical problem for health care systems internationally. It is important to detect patients suspected of infection quickly and accurately. Urgent solutions are required to better detect and prevent the spread of the virus. Nanobiosensors have the greatest potential for detecting and, thus, preventing the spread of the coronavirus pandemic. Nanomaterials have a great impact when used in biosensing applications because of their unique properties at nanoscale. Thus, the fabrication of nanobiosensors can provide the tools necessary to perform diagnosis in a few seconds, with high precision for mass screening. Many proposed nanobiosensors for COVID-19 causative virus detection are in the pipeline for growth, and are in various stages of development. In this review, an up-to-date overview is provided to discuss the most current contributions of biosensors designed to detect respiratory viruses that cause epidemics and pandemics, and to compare them, in terms of detection mechanisms, significance of biological receptors, and surface modifications; the challenges and trends in the field are also discussed. The structural protein components of influenza viruses and coronaviruses, responsible for the mechanisms by which these respiratory viruses attack and replicate were also taken into account, emphasizing the role, classification, and characteristics of nanobiosensors for human coronavirus detection, especially for the COVID-19 disease pandemic.

## Figures and Tables

**Table 1:** The animal origins of various coronaviruses, the corresponding diseases and some characteristics of them in comparison with influenza viruses ^a^.

Disease	Flu (swine flu)	SARS 2002	MERS 2012	COVID 19
Virus Name	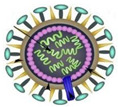 H1N1 Influenza A	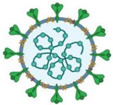 SARS–CoV-1 or SARS-CoV	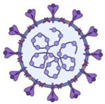 MERS-CoV	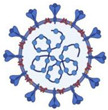 SARS–CoV-2 or 2019-nCoV
Origin of Virus	Bird Influenza A	SARS-like BAT-CoV	SARS-like BAT-CoV	BaT-CoV RaTG13 or Pangolin-CoV
Intermediate host	Pig Influenza A	Civet Cat	Camel	Pangolin
Incubation period	1–4 days	2–7 days	5 days	2–14 days
Symptoms	Fever, cough, shortness of breath or difficulty breathing, mild respiratory tract infections, sometimes severe and acute diarrhea and acute vomiting	Fever, cough, shortness of breath or difficulty breathing, severe acute respiratory syndrome, 10 % mortality rate	Fever, cough, shortness of breath or difficulty breathing, severe acute respiratory syndrome, 37% mortality rate	Fever, cough, shortness of breath or difficulty breathing, severe acute respiratory syndrome; mortality rate; diarrhea, fatigue, vomiting, muscle or body aches, headache, loss of the sense of smell or taste.

^a^ Adapted from: [12,63,64,65].

**Table 2:** List of laboratory tests recommended with common clinical indicators associated with the COVID-19 tests for adult patients.

Biomarkers	Normal Patient	Infected Patient	Severe Conditions	Ref.
Serum ferritin (ng/mL)	15.0–150.0	800.4 (452.9–1451.6)	Inflammation	[90]
C-reactive protein (mg/mL)	0.0–1.0	57.9 (20.9–103.2)	Viral infection	[90]
Interleukin-2R (U/mL)	223.0–710.0	757.0 (528.5–1136.3)	High plasma concertation	[90,91]
Cytokines (IL-6) (pg/mL)	0.0–7.0	7.9	Syndrome related to “cytokine storm”	[92]
D-Dimer (µg/mL)	0–0.243	0.5	Abnormal blood coagulation distributed coagulopathy	[93]
Serum amyloid A (SAA) (mg/L)	0–10	108.4	Inflammation	[93]

**Table 3:** Electrochemical based nanobiosensors of epidemic/pandemic influenza viruses in comparison with coronaviruses (SARS/MERS-CoV and 2019-nCoV).

Biological Samples	Nanomaterials	Detection Methods	Target	Limit of Detection (LOD) Linear Range (LR)		Ref.
**INFLUENZA VIRUSES**						
Biological substances such as DNA and proteins	Graphene (single-layer hexagonal carbon networks)	Field-Effect Transistor (FET)	Lectin	LOD: 130 pM LR: –		[110]
Oligonucleotide sequences derived from an H5N1 avian influenza	rGO reduced graphene oxide	Field-Effect Transistor (FET)	Gene (H5N1)	LOD: 50 pM LR: –		[111]
Saliva	Nanocrystalline boron-doped diamond	Electrochemical impedance (EIS)	Influenza virus M1 protein	LOD: 5 × 10^−14^ g/mL LR: –		[86]
Egg sample	Graphene gold hybrid nanocomposite	Electrochemical impedance EIS)	Influenza A virus	LOD: 10^–8^ U/mL LR: 10^−8^–10^−10^ U/mL		[124]
Fetal bovine serum, extraneous bovine serum albumin (BSA)	Gold electrode	Electrochemical impedance spectroscopy (EIS)	Human influenza virus type A (H3N2)	LOD: 8 ng/mL LR:		[112]
Saliva buffer	Diamond biosensor (nano-scale boron-doped diamond surface sensor)	Electrochemical impedance spectroscopy (EIS)	M1 protein of influenza A virus	LOD: 1 fg/mL LR: 1–100 fg/mL		[113]
Samples contained bovine serum albumin solution (BSA (0.5%)	Nanostructured hybrid bilayers on gold electrodes	Electrochemical impedance spectroscopy (EIS)	Human influenza A virus (H1N1)	LOD: 0.024 μg/mL LR: –		[78]
Viral sample of inactivated, but intact influenza viruses H3N2	Gold electrode	Electrochemical impedance spectroscopy (EIS)	Human influenza A virus (H3N2)	LOD: 1.3 × 10^4^ viruses/mL LR: –		[114]
Isolated AIV H5N1 sample incubated for 45 min at 37 °C	Magnetic iron oxide (Fe3O4) nanobeads	Electrochemical impedance spectroscopy (EIS)	Avian influenza virus (AIV) (H5N1)	LOD: 0.0128 HA unit/50 μL. LR: –		[116]
Biological samples with avian influenza virus	Magnetic streptavidin-coated 30 nm nanobeads	Electrochemical impedance spectroscopy (EIS)	Avian influenza virus (AIV) (H5N1)	LOD: 103 EID 50/mL LR: –		[115]
Inactivated avian influenza virus H5N1 sample	Concanavalin A-glucose oxidase-Au nanoparticles (ConA-GOx-AuNPs)	Electrochemical impedance spectroscopy (EIS)	Avian influenza virus (AIV) (H5N1)	LOD: 0.04 HAU/mL LR: –		[117]
Commercial sample Spike saliva	Gold paper electrode	Electrochemical Impedance spectroscopy (EIS)	H1N1 antigen	LOD: 4.70 PFU/mL LR: –		[125]
Influenza viral particles in infected swine nasal samples	Reduced graphene oxide nanosheets (RGO)	Chronoamperometry (CA)	Human influenza A virus (H1N1)	LOD: 0.5 PFU/mL LR: 1 to 10^4^ PFU/mL		[118]
Virus culture in embryonated chicken egg	Gold nanoparticles (AuNPs)	Chronoamperometry (CA)	Influenza virus (H9N2)	LOD: 16 HAU LR: –		[119]
Virus samples in chicken embryo cultures	Conducting polymer of PEDOT-poly (3,4-ethylene-dioxythiophene) PSS film	Amperometry	Human influenza A virus (H1N1)	LOD: 0.025 HAU LR: –		[126]
Commercial ELISA kits Probe sequence (avidin from egg whites)	ZnO-NRs Glass Wafer/PD MS	Amperometry	(H1N1), (H5N1), and (H7N9) influenza	LOD: 1.00 pg/mL LR: 1–10 ng/mL		[27]
Throat swab samples	Gold electrode	Amperometry	Tetrahedral DNA probe of the H7N9/ssDNA of H7N9	LOD: 0.750 pM LR: –		[127]
Analyte samples collected from the throats of live animals, fecal content, and blood	Graphene oxide (GO) nanostructures Dual carbon SPE	Chronoamperometry and Differential pulse voltammetry (DPV)	HA proteins of Influenza virus (H5N1)/(H1N1)	LOD: 9.4 pM (Commercial H1N1) LOD: 8.3 pM (Commercial H5N1) LR: 25–100 pM		[121]
Nasal swab and oropharyngeal samples	Gold screen printed electrode (SPE)	Cyclic voltammetry (CV)	ss-cDNA of the H1N1	LOD: 0.004 ng in 6 µL		[128]
Chicken serum	Gold electrode	Cyclic voltammetry (CV)	HA protein of H5N1	LOD: 1.00 pM		[129]
Negative chicken swab samples	Fe3O4 Magnetic Nanoparticles	Cyclic voltammetry (CV)	Avian influenza virus (AIV) (H5N1)	LOD: 0.367 HAU/mL LR: 0.0025 to 0.16 HAU		[120]
Human blood, nasal swab, saliva, and urine	AP-Neu5Ac substrate	Cyclic Voltammetry (CV) Linear sweep Voltammetry (LSV)	Viral surface of NA-neuraminidase	LOD: 5.6 ng/mL LR: 0–900 nG/mL		[108]
Cell culture and viral infection cells	Specific anti-PB1-F2 antibody on the surface of the Au micro-electrode modified with polypyrrole bearing ferrocene	Cyclic Voltammetry (CV) Differential pulse voltammetry (DPV)	Protein of influenza A virus (PB1-F2)	LOD: 0.42 nM LR: –		[87]
Human serum	Pt NPs_porous ZnO spheres	Voltammetric (Cyclic Voltammetry)	DNA sequence of influenza virus	LOD: 0.76 pg/mL LR: 0.001–60 ng/mL		[122]
Diluted human serum samples spiked	AuNPs	Differential pulse voltammetry (DPV)	H5N1 DNA aptamer/antiH5N1	LOD: 100 fM LR: 100 fM–10 pM		[130]
Saliva from a healthy person	Superhydrophobic paper/conductive carbon paste	Differential pulse voltammetry (DPV)	H1N1 antibody/H1N1 antigen	113 PFU/mL		[131]
Antibodies from Hen sera from individuals vaccinated and non-vaccinated	Gold electrode	Osteryoung square wave voltammetry (OSWV)	His6-H5 HA/antiH5N1	LOD: 2.40 pg/mL LR: 4.0–100.0 pg/mL		[132]
Diluted human and swine serum Vaccinated mice sera	Gold electrode	Osteryoung square wave voltammetry (OSWV).	His6-H1HA/anti-H1N1	LOD: – LR: –		[133]
Biological samples	Gold electrode	Osteryoung Square Wave Voltammetry (OSWV)	ssDNA of H5N1/ RNA of the H5N1	LOD: 3.00 copies/µL		[134]
The probe DNA (avidin-biotinylated probe DNA)	AuNPs	Voltammetric	Influenza virus type A (H1N1)	LOD: – LR: –		[135]
Real patient samples	CdS quantum dots (QDs)	Voltammetric	Influenza virus	LOD: 0.06 mM LR: 0.06–0.5 mM		[53]
Infected swine nasal samples	Single walled carbon nanotubes	Conductometry	Swine influenza virus (SIV) (H1N1)	LOD: 180 TCID50/mL LR: –		[123]
Clinical exhaled breath condensate (EBC) samples	Silicon nanowire (SiNW)	Conductometry	Human influenza A viruses (H1N1) and (H3N2)	LOD: 2.9 viruses/µL LR: –		[55]
Saliva from a healthy person	Superhydrophobic paper/conductive carbon paste	Differential pulse voltammetry (DPV)	H1N1 antibody/H1N1 antigen	113 PFU/mL		[131]
Antibodies from Hen sera from individuals vaccinated and non-vaccinated	Gold electrode	Osteryoung square wave voltammetry (OSWV)	His6-H5 HA/antiH5N1	LOD: 2.40 pg/mL LR: 4.0–100.0 pg/mL		[132]
**CORONAVIRUSES**						
**SARS-CoV**						
Streptavidin (S-Av) analyte	Single-walled carbon nanotubes (SWCNTs)	Field-Effect Transistor (FET)	nucleocapsid (N) protein of the SARS virus	LOD: physiological conditions	[136]	
Bovine serum albumin	In_2_O_3_ nanowire with an AMP (Fibronectin, Fn)	Field-Effect Transistor (FET)	SARS Virus N-Protein Nucleocapsid (N)	LOD: sub-nanomolar Concentration of N protein	[45]	
A 30-mer sequence of SARS Virus	100 nm sputtered gold film	Cyclic voltammetry (CV)	SARS virus sequence	LOD: 7 × 10^−6^ M LR. 10^−5^ to 5 × 10^−4^ M	[137]	
Bovine serum albumin (BSA) and a rabbit immuno- Globulin G (RIgG) labeled with aurothiomalate	Au electrodeposition on glassy carbon electrodes (GCEs	Cyclic voltammetry (CV)	30-mer sequence of the SARS virus	LOD: 15 fmol (30 μL) LR: –	[138]	
DNA sequence of SARS virus	Gold nanoparticles	Cyclic voltammetry (CV) Screen-printed carbon electrodes (SPCEs)	SARS virus sequence	LOD: 2.5 pmol/L LR: –	[139]	
Clinical specimens	Au@Fe3O4 nanocomposite Fe3O4 NPs (for premix A prep a-ration) and graphene oxide (GO) (for premix B preparation)	Differential pulse voltammetry (DPV) with a smartphone	RNA of SARS-CoV-2	LOD: 200 copies/mL LR: –	[140]	
Protein sample of SARS-CoV	-	high electron mobility transistors (HEMTs	SARS-CoV nucleocapsid protein	LOD: 0.003 nM LR: –	[99]	
**MERS-CoV or hCoV-EMC/2012**						
Spiked nasal samples	AuNPs on carbon electrode	Square wave voltammetry (SWV)	Middle East respiratory syndrome coronavirus (MERS-CoV) and human coronavirus (hCoV)	LOD: 0.4 pg/mL LR: –	[62]	
**2019-nCoV or SARS-CoV-2**						
Human Nasopharyngeal Swab Specimens, from COVID-19 patients Cultured virus	Graphene sheet	Field-Effect Transistor (FET)	SARS-CoV-2 spike protein	LOD: 1.6 × 10^1^ pfu/mL in culture medium 2.42 × 10^2^ copies/mL in clinical samples LR: –	[69]	
Green Monkey Kidney Cell Culture	Membrane-Engineered Vero Cells (Vero/Anti-S1)	Bioelectric Recognition Assay (BERA)	SARS-CoV-2 S1 Spike Protein Antigen	LOD: 1 fg/mL LR: –	[4]	
Spiked saliva samples	Fluorine doped tin oxide (FTO) electrode with gold nanoparticle (AuNPs)	Cyclic Voltammetry (CV), Differential Pulse Voltammetry (DPV)	nCovid-19 spike antigen (nCovid-19Ag)	LOD: 90 fM with eCovSens and 120 fM with potentiostat (spiked saliva samples LOD: 10 fM (in-house built device) of nCovid-19 Ag LR: 1 fM to 1 μM in standard buffer	[141]	

**Table 4:** Optical detection technique, and their properties of human coronaviruses, including recently developed 2019-nCoV nanobiosensors and influenza viruses.

Biological Samples	Nanomaterials	Detection Methods	Target	Limit of Detection (LOD) Linear Range (LR)	Ref.
**INFLUENZA VIRUSES**					
H5N1 virus in biological samples	Gold nanoparticles (AuNPs)	Localized surface plasmon resonance (LSPR); Colorimetric	H5N1 virus	LOD: 0.086 mU/mL LR: 0.1–5 mU/mL	[165]
Viral strains, tracheal samples	Optical SPR fiber sensor	Surface plasmon resonance (SPR)	Avian Influenza virus	LOD: 5.14 × 10^5^ EID_50_/0.1mL LR: –	[166]
H5N1–infected feces samples	Gold chip	Surface plasmon resonance (SPR)	H5N1 aptamer/H5N1 whole virus	LOD: 200 EID_50_/mL LR: –	[167]
Infected cells A549 type with wild type virus or with its PB1-F2 knock-out mutant	Immobilization of anti-PB1-F2 anti-body on the surface of Au micro-electrode modified with polypyrrole bearing ferrocene	Surface Plasmon Resonance (SPR)	PB1-F2 protein of influenza A virus	LOD: 0.42 nM LR: –	[87]
Biomolecular samples	Gold sensor	Surface plasmon resonance (SPR)	H5N1 antigen/H5N1 antibody ssDNA of the H1N1	LOD: 193.3 ng/mL LR: –	[168]
Blood samples	Gold binding polypeptide (GBP)–fusion protein	Localized surface plasmon resonance/SPR imaging (LSPR/SPRi)	Influenza B virus	LOD: 1 pg/mL LR: –	[77]
Chicken serum	Au spike-like nanoparticle (hAuSN) immobilized on the indium-tin-oxide (ITO) substrate	Localized surface plasmon resonance (LSPR)	HA protein from H5N1	LOD: 1.00 pM LR: –	[169]
Nasal mucosa from flulike syndrome patients	Gold chip	Intensity-modulated surface plasmon resonance (IM-SPR)	Attenuated reassorted H7N9 antigen	402 copies/mL	[170]
Clinically isolated virus type H3N2	Antibody-Gold nanoparticles	Fluorescence localized surface plasmon resonance (FL-LSPR)	H3N2 Virus	LOD: 10 PFU/mL LR: –	[30]
Human serum	DNA triplex with berberine	Fluorescence-fluorescein isothiocyanate assay (FL/FICT)	Gene of H7N9 virus DNA	LOD: 0.14 nM LR: –	[171]
Biological tissue	Quintenary alloyed CdZnSeTeS quantum dots	Near-infrared (NIR) Fluorescence	RNA sequence of influenza virus	LOD: 1 copy/mL LR: 0–14 copies/mL	[52]
Commercial H5N1–Human serum	Ag@SiO2 NPs	Fluorescence	H5N1 aptamer/Recombinant HA protein of H5N1	LOD: 2.00–3.5 ng/mL LR: –	[172]
Human serum samples	Liposome-based sensor	Spectrophotometry	Influenza virus H5N1 based on enzyme encapsulated liposome	LOD: 0.04 ng/mL LR: 0.1–4.0 ng/mL	[173]
Tracheal swabs collected from wild birds	Polydiacetylene (PDA) vesicles	UV-VIS spectrometer	H5N1 antibody/HA of the H5N1	LOD: 0.530 copies/µL LR: –	[174]
-	Gold nanoparticles (AuNPs) modified with monoclonal anti-hemagglutinin antibody (mAb).	Colorimetric immunosensor	Influenza A virus (IAV)	LOD: 7.8 hemagglutination units (HAU) LR: –	[175]
Viral culture	Gold nanoparticles (AuNPs)	Surface enhanced Raman scattering (SERS)-based lateral flow assay (LFA)	Viral particles (VP)	LOD: 1.9 × 10^4^ PFU/mL LR: 0–1.0 × 10^6^ PFU/mL	[176]
Viral nucleic acid	BaGdF 5: Yb/Er upconversion nanoparticles (UCNPs) to AuNPs	Luminescence Resonance Energy Transfer (LRET)	H7 hemagglutinin gene sequence	LOD: 7 pM LR: 10 pM to 10 nM	[177]
**CORONAVIRUSES**					
**SARS-CoV**					
Human serum Bovine serum albumin (BSA)	Gold nanoparticles	Localized surface plasmon coupled fluorescence (LSPCF) fiber-optic	SARS-CoV nucleocapsid protein (N protein)	LOD: 1 pg/mL LR: –	[163]
Rabbit anti-SCVme	Gold micropatterned chip	Surface plasmon resonance (SPR)	GBP-E-SCVme (SARS-CoV) fusion proteins/anti-SCVme	LOD: 0.200 µg/mL LR: –	[178]
Protein sample	–	Surface plasmon resonance (SPR) Fluorescence resonance energy transfer (FRET)	SARS-CoV genome sequence (full- length and N-terminal residues 1–7 deleted SARS 3Clpros)	LOD: – LR: –	[67]
Culture sample of SARS protein, enhanced GFP-green fluorescent protein and RFP-red fluorescent protein	poly(hydroxyalkanoate) (PHA) microbead	Fluorescence Flow cytometry	SARS-CoV envelope gene sequence	LOD: – LR: –	[179]
Vero E6 Cells	Green fluorescent protein (GFP)	Fluorescence	The 3a gene encodes a non-structural viral protein of SARS-Coronavirus	LOD. – LR: –	[180]
Protein sample	-	Fluorescence resonance energy transfer (FRET)	SARS coronavirus NTPase/Helicase	LOD: – LR: –	[181]
Upper-strand DNA and fluorescent-dye-conjugated bottom-strand DNA	Graphene oxide (GO) sheet	Fluorescence	SARS-CoV helicase	LOD: – LR: –	[182]
Lung samples cell (A549 human alveolar epithelial cells or inner medullary collecting duct (IMCD) mouse kidney epithelial cells taken after 6 days of infection with SARS-CoV)	-	Flow cytometry Affinity chromatography for purification of Spike-Fc protein)	SARS-CoV Spike Fc protein	LOD: – LR: –	[9]
Control samples Unlabeled nucleic acids in solution	-	Flow cytometry based on fluorescence	SARS-hCoV-M SARS-hCoV-N parainfluenza virus type 3(PIV-3), respiratory syncytial virus (RSV)	LOD: 26 fmol at an mean fluorescence intensity (MFI) of 5.7 (for SARS-N) LOD: 37 fmol (for SARS-M, hepatitis C virus , parainfluenza virus type 3, RSV) LR.26–56 fmol for SARS-M, HCV, PIV-3, RSV).	[183]
Serum samples (B cells of SARS convalescent patients; whole inactivated SARS-CoV virions)		Imaging ellipsometry (Real-time spectroscopic ellipsometry detect the protein layer pattern on the microarray surface.	B cells of SARS whole inactivated SARS-CoV virions	LOD: – LR: –	[184]
Human serum from healthy donor Synthetic RNA aptamer	QDs-conjugated RNA aptamer On glass CHIP	optical QDs-based RNA aptamer chip	SARS-CoV N protein	LOD: concentrations as low as 0.1 pg/mL	[98]
genomic DNA	Functionalized Photonic Nanocrystals	Optical detection	SARS coronavirus antigenic surface protein		[185]
Rabbit anti-SARS coronavirus surface antigen (Rabbit anti SCVme)	Gold micropatterned chip	Surface plasmon resonance (SPR)	SARS coronavirus surface antigen (SCVme)	LOD: 0.200 µg/mL LR: –	[178]
Serum samples (B cells of SARS convalescent patients; whole inactivated SARS-CoV virions)		Imaging ellipsometry (real-time spectroscopic ellipsometry detects the protein layer pattern on the microarray surface)	B cells of SARS whole inactivated SARS-CoV virions (scFv, b1 and h12 molecule)	LOD:2.2 µg/mL (b1) and 34 µg/mL (h12) LR: -	[184]
Human serum from healthy donor Synthetic RNA aptamer	QDs-conjugated RNA aptamer On glass chip	Optical QDs-based RNA aptamer chip (Confocal laser scanning microscopy)	SARS-CoV N protein	LOD: concentrations as low as 0.1 pg ml^−1^	[98]
**MERS-CoV or hCoV-EMC/2012**					
Clinical sample	Gold nanoparticles (AuNPs)	Localized surface plasmon resonance (LSPR); Colorimetric assay	MERS-CoV DNA samples	LOD: 1 pmol/µL LR: –	[186]
Convalescent patient serum clinical isolate hCoV-EMC/2012 from green monkey kidney (Vero B4) cells	-	Immunofluorescence microscopy	hCoV-EMC/2012 (MERS-CoV)	LOD: 4.1 RNA copies/reaction	[187]
Synthetic DNA oligonucleotides samples	Silver nanoparticles (AgNPs)	Colorimetric assay	MERS-CoV DNA	LOD: 1.53 nM	[188]
**2019-nCoV or SARS-CoV-2**					
Respiratory secretions upper respiratory tract (URT) specimen	Gold nanoislands functionalized (AuNIs) with complementary DNA receptors	Plasmonic photo-thermal (PPT) and localized surface plasmon resonance (LSPR)	SARS-CoV-2 Nucleic acid	LOD: 0.22 pM LR: –	[1]
Clinical samples	-	Fluorescent detection	SARS-CoV-2 RNA	LOD: 2 copies per sample	[189]
Isolated RNA samples	Gold nanoparticles	Colorimetric assay	RNA sequence of SARS-CoV-2	LOD: 0.18 ng/µL of RNA Dynamic range: 0.2–3 ng/µL.	[190]
Blood samples collected from 397 PCR confirmed COVID-19 patients and 128 negative patients	gold nanoparticle (AuNP) colloids	colorimetric assay	SARS-CoV-2 IgG-IgM combined antibody	LOD: – LR: –	[191]

**Table 5:** Magneto-optical detection method and their parameters for human coronaviruses including COVID-19 causative virus and influenza viruses.

Biological Samples	Nano-/Micro Materials	Detection Methods	Target	Limit of Detection (LOD) Linear Range (LR)	Ref.
**INFLUENZA VIRUSES**					
Virus samples in aqueous buffer and human serum	Ag@SiO_2_ nanoparticles	Metal enhanced fluorescence (MEF)	Influenza H5N1	LOD: 3.5 ng/mL LR: 2–200 ng/mL	[172]
Clinical virus in complex biological samples	Au/Fe_3_O_4_ decorated graphene	Fluorescence	Influenza H1N1	LOD: 7.27 fg/mL LR: 10–10^4^ fg/mL	[209]
Complex biological samples	Au/iron oxides (Au/IONPs)-decorated graphene	Magnetofluoro immunoassay (Plasmonic-magnetic graphene platform for virus detection)	Influenza H1N1 In serum	LOD: 6.07 pg/mL LR: –	[209]
Clinically isolated human serum samples	Silica-shelled magnetic nanobeads (MagNBs) and gold nanoparticles	Magnetic nano(e)zyme-linked immunosorbent assay (MagLISA)	Influenza virus A	LOD: 5 × 10^−12^ g/mL (by human eyes) LOD: 44.2 × 10^−15^ g/mL (by a microplate reader) LR: 5 × 10^−15^–5 × 10^−6^ g/mL	[210]
**CORONAVIRUSES**					
**SARS/MERS-CoV**					
Paired human sera and control serum samples for each hCoV	Multiplexed magnetic microsphere	MMIA- multiplexed magnetic microsphere immunoassay Fluorescence	SARS-CoV and MERS-CoV Immunoglobulin G antibodies specific for recombinant nucleocapsid proteins (from SARS-CoV, and MERS-CoV, hCoVs, 229E, NL63, OC43, HKU1	LOD: – LR: –	[211]
**2019-nCoV or SARS-CoV-2**					
SARS-CoV-2 pseudovirus in 200 μL serum samples	Poly (amino ester) with carboxyl groups (PC)-coated magnetic nanoparticle (pcMNPs)	Fluorescence and convectional RT-PCR protocol	Viral RNA extraction of SARS-CoV-2	LOD: 10 copies of pseudovirus	[212]
Fetal bovine serum (FBS)	Magnetic nanoparticle (MNP)	Optomagnetic sensing	SARS-CoV-2 RdRp coding sequences	LOD: 0.4 fM dynamic Detection range: 3 orders of magnitude and a total assay time of ca. 100 min	[14]

**Table 6:** Mass sensitive detection method and their parameters for developed SARS-CoV and influenza virus nanobiosensors.

Biological Samples	Nanomaterials	Detection Methods	Target	Limit of Detection (LOD) Linear Range (LR)	Ref.
**INFLUENZA VIRUSES**					
-	Gold film	Quartz crystal microbalance (QCM)	Hemagglutinin (HA) protein of influenza A virus	LOD: 4.7 × 10^−2^ µM, (0.26 µg/mL) LR: –	[235]
Sample of human influenza A virus (H1N1) incubated in a chicken egg culture	Poly(EDOT-co- EDOTOA) Films	Quartz crystal microbalance (QCM)	Human influenza A virus H1N1	LOD: 0.012 HAU LR: –	[236]
Influenza A virus (VR-544, H3N2) samples	QCM electrodes	Quartz crystal microbalance (QCM)	Influenza A virions, influenza H3N2 polyclonal IgG	LOD: 4 virus particles/mL	[237]
Commercial H5N3	Lead zirconate titanate (PZT) piezoelectric disc	Piezoelectric–SPM	H5N3 surface glycoprotein	105 vp/mL (100 µm thick)	[228]
Biological sample	–	Surface acoustic wave (SAW)	HA proteins of Influenza A virus sub type H1N1	LOD: 1 ng /mL LR: –	[238]
commercial H5N3	Lead zirconate titanate (PZT) piezoelectric disc	Piezoelectric—SPM	3′SLPAA polymer/H5N3 surface glycoprotein	10^5^ vp/mL (100 µm thick)	[228]
**CORONAVIRUSES**					
**SARS-CoV**					
Sputum	PZ crystal surface	Immunoassay	SARS-CoV	LOD: 0.6 µg/mL LR: –	[102]
Biological sample	Piezoelectric immunosensor	Quartz crystal microbalance (QCM)	SARS-CoV spike protein S1	–	[239]
High protein sera sample	Aptamer coated paramagnetic nanoparticles	Piezoelectric	SARS-CoV helicase protein	LOD: 3.5 ng/mL	[240]
**2019-nCoV or SARS-CoV-2**					
Oral swab samples	Nanoparticles	Quartz crystal microbalance (QCM)	SARS-CoV-2 spike protein	–	[241]
